# The ubiquitin ligase UBR4 and the deubiquitylase USP5 modulate the stability of DNA mismatch repair protein MLH1

**DOI:** 10.1016/j.jbc.2024.107592

**Published:** 2024-07-18

**Authors:** Chenyu Mao, Siqi Li, Jun Che, Dongzhou Liu, Xinliang Mao, Hai Rao

**Affiliations:** 1 Department of Biochemistry, School of Medicine, Southern University of Science and Technology, Shenzhen, China; 2 Department of Rheumatology and Immunology, Shenzhen People’s Hospital, Shenzhen, Guangdong, China; 3 The First Affiliated Hospital, Southern University of Science and Technology, Shenzhen, Guangdong, China; 4 Guangdong Provincial Key Laboratory of Protein Modification and Degradation, School of Basic Medical Sciences, Guangzhou Medical University, Guangzhou, Guangdong, China; 5 Key University Laboratory of Metabolism and Health of Guangdong, Southern University of Science and Technology, Shenzhen, China

**Keywords:** DNA mismatch repair, MLH1, E3 ligase, deubiquitylase, ubiquitin

## Abstract

MLH1 plays a critical role in DNA mismatch repair and genome maintenance. MLH1 deficiency promotes cancer development and progression, but the mechanism underlying MLH1 regulation remains enigmatic. In this study, we demonstrated that MLH1 protein is degraded by the ubiquitin-proteasome system and have identified vital cis-elements and trans-factors involved in MLH1 turnover. We found that the region encompassing the amino acids 516 to 650 is crucial for MLH1 degradation. The mismatch repair protein PMS2 may shield MLH1 from degradation as it binds to the MLH1 segment key to its turnover. Furthermore, we have identified the E3 ubiquitin ligase UBR4 and the deubiquitylase USP5, which oppositely modulate MLH1 stability. In consistence, UBR4 or USP5 deficiency affects the cellular response to nucleotide analog 6-TG, supporting their roles in regulating mismatch repair. Our study has revealed important insights into the regulatory mechanisms underlying MLH1 proteolysis, critical to DNA mismatch repair related diseases.

DNA mismatch repair (MMR) is an evolutionarily conserved mechanism that corrects DNA mismatches, which are often the results of DNA injuries (*e.g.*, methylation, oxidation, inter-strand crosslinks) or the mistakes made by DNA polymerases during DNA replication (1, 2, 3). MMR inactivation leads to highly elevated mutation rates, microsatellite instability and a solid predisposition to many types of cancers, including colorectal, endometrial, brain, hematological, and prostate cancers (4, 5).

In eukaryotic cells, MMR requires the MutS and MutL complexes (6, 7, 8, 9), which are presented as hetero-dimers. MutSα (*i.e.*, the MSH2-MSH6 dimer) or MutSβ (*i.e.*, the MSH2-MSH3 dimer) recognize the base-base mismatches or the insertions-deletions loops, respectively. MutS subsequently recruits MutL, mostly MutLα (*i.e.*, the MLH1-PMS2 complex), which induces a nick near the DNA lesion. The nick provides the entry point for a DNA helicase and one of a few exonucleases, such as exonuclease1 (EXO1), to degrade the DNA strand containing the mismatch. The resulting ssDNA gap is then resynthesized by DNA polymerase δ, assisted by proliferating cell nuclear antigen (PCNA), replication factor C (RFC), and replication protein A (RPA), and finally re-ligated by DNA Ligase I (3, 6, 7, 10). The other major MutL complex is MutLγ (*i.e.*, the MLH1-MLH3 complex), which has a crucial role in meiotic homologous recombination and a minor role in removing insertion-deletion loops along with MutSβ (the MSH2-MSH3 dimer) (11, 12, 13). MMR is a highly coordinated process with these proteins working concertedly, allowing precise removal of DNA mispairing or damaged DNA.

Various genetic and epigenetic alterations have been found in MMR genes in cancers (5, 14, 15). MMR proteins are also modulated at the post-translational levels (16, 17, 18, 19, 20). Given its central role in MMR, MLH1 might also be highly regulated. Interestingly, over-expression of either MLH1 or PMS2 leads to defective MMR and hyper-mutagenesis in budding yeast cells, while co-expression of its partner can alleviate the highly increased mutations (21). It is, therefore, interesting to investigate if MLH1 is regulated at the post-translational level.

In the present study, we found that MLH1 protein stability is regulated by the ubiquitin-proteasome system. Its partner PMS2 is the key to maintaining MLH1 stability. We also find that EXO1 can influence the stability of MLH1. We revealed that the ubiquitin E3 ligase UBR4 and the deubiquitylase USP5 modulate the ubiquitylation and degradation of MLH1. Our results provide a novel entry to understanding the proteolytic regulation of MMR- and MLH1-related disorders.

## Results

### MLH1 is degraded by the ubiquitin-proteasome system

**Figure 1:**
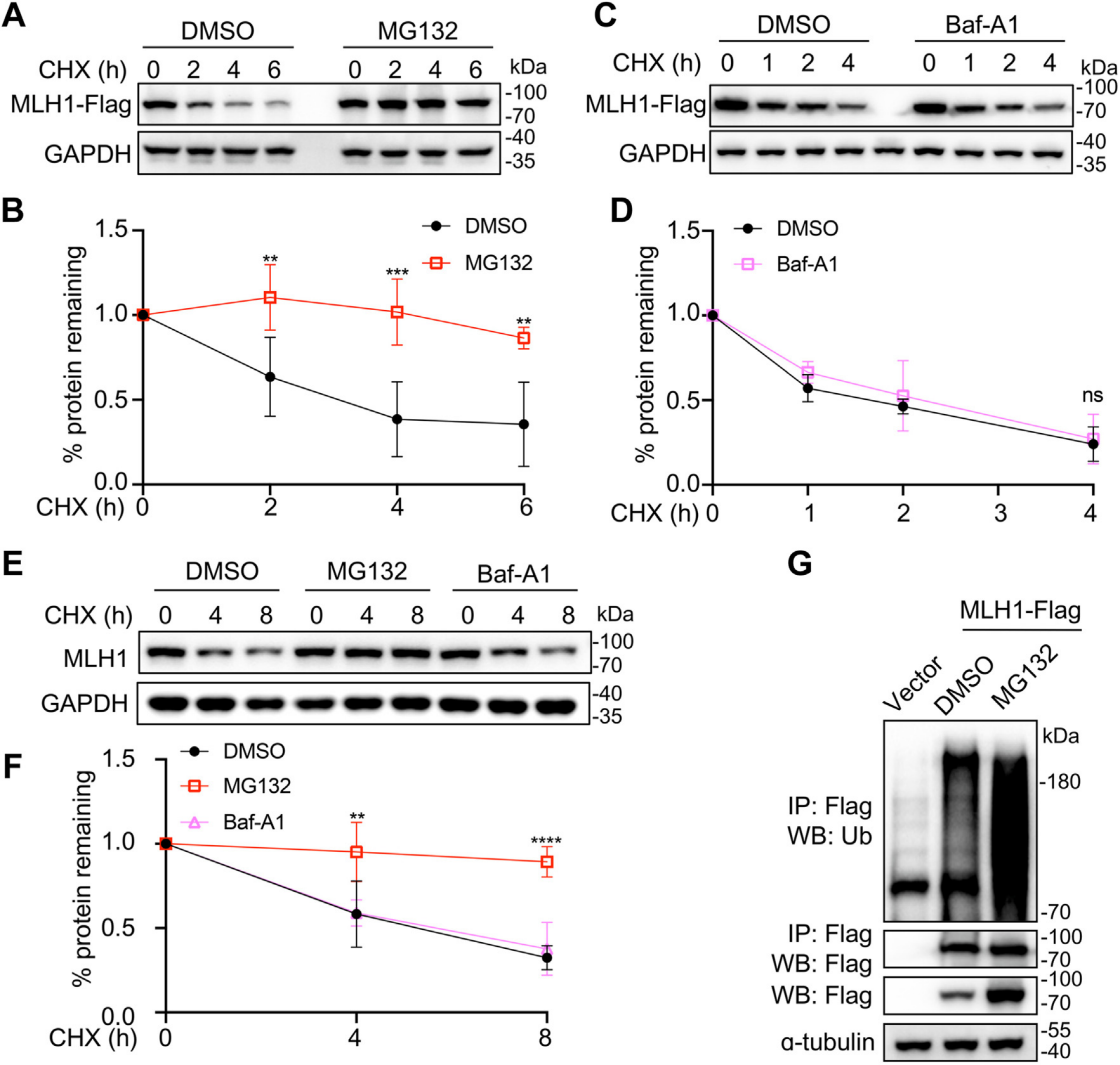
**MLH1 is degraded *via* the ubiquitin-proteasome pathway.***A*, ectopically expressed MLH1 turnover is blocked upon the treatment of MG132, a proteasome inhibitor. HEK-293T cells were transfected with the MLH1-Flag plasmid for 48 h, followed by the MG132 treatment, and the samples were collected for Western blotting analysis. *B*, quantitation of the data in *A* (means ± SD in three independent experiments, *∗∗p* < 0.01, *∗∗∗p* < 0.001, two-tailed Student’s *t* test). *C*, MLH1 degradation is independent of the lysosome. HEK-293T cells bearing MLH1-Flag were treated with or without Baf-A1, and the degradation rate of MLH1 was monitored. The empty vector serves as a negtive control. *D*, quantitation of the data in *C* (means ± SD in three independent experiments, ns > 0.05, two-tailed Student’s *t* test). *E*, endogenous MLH1 degradation requires the proteasome. HeLa cells were treated with MG132 or Baf-A1 for indicated periods. Cells were then collected to monitor MLH1 stability. *F*, quantification of the data in *E* (means ± SD in three independent experiments, *∗∗p < 0.01*, *∗∗∗∗p* < 0.0001, two-tailed Student’s *t* test). *G*, MLH1 is modified by ubiquitin. HEK-293T cells were transfected with MLH1 plasmids for 48 h, followed by the MG132 treatment. The cell lysates were then prepared for immunoprecipitation, and Western blotting analysis indicated the MLH1 ubiquitylation profile. 15% of MG132-treated samples were added to ensure the amount of MLH1 in DMSO or MG132 samples was equal for a better comparison of the ubiquitylation pattern.

Excessive MLH1 leads to defective MMR and hyper-mutations in yeast cells, so we suspected that the MLH1 level may be kept in check by proteolysis in higher eukaryotic organisms. To evaluate whether MLH1 is degraded, HEK-293 cells expressing Flag-tagged MLH1 were treated with cycloheximide (CHX), a protein synthesis inhibitor (22), and collected at various time points. We found MLH1 is degraded (Fig. 1*A*). The proteasome and lysosome are two major proteolytic systems in eukaryotes. We then treated cells expressing MLH1-Flag with MG132, a proteasome inhibitor (23), or bafilomycin A1 (Baf-A1), an inhibitor of lysosomes (24). Whereas MG132 drastically blocked MLH1 degradation (Fig. 1, *A* and *B*), there was no significant change in MLH1 degradation rate in Baf-A1-treated cells compared with control cells (Fig. 1, *C* and *D*). Endogenous MLH1 in HeLa cells was found to be degraded in a proteasome-dependent manner as well (Fig. 1, *E* and *F*). We also monitored the ubiquitylation pattern of MLH1 and found MLH1 ubiquitylation is enriched upon the MG132 treatment (Fig. 1*G*). These results suggest that the MLH1 is degraded *via* the ubiquitin-proteasome system.

### The amino acids 516 to 650 region is essential for MLH1 degradation

**Figure 2:**
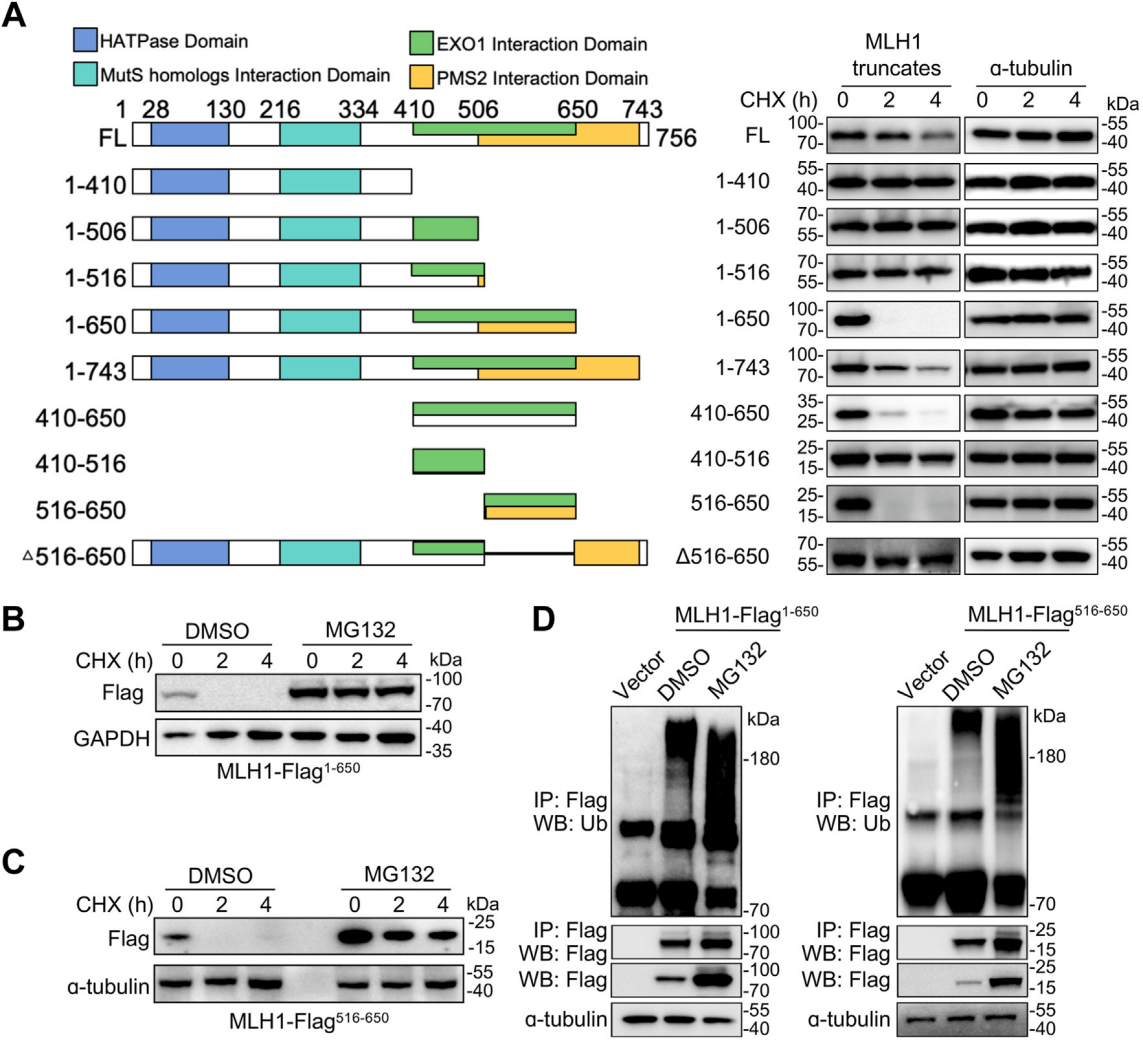
**Identification of the sequences essential for MLH1 degradation.***A*, analysis of various MLH1 mutants. Serials of Flag-tagged MLH1 truncations were constructed as indicated and transfected into HEK-293T cells. Cells were then collected after the CHX treatment, and the cell lysates at indicated time points were prepared for the Western blotting analysis for MLH1-Flag fragments. *B* and *C*, MLH1 fragments are mainly degraded by the proteasome. The plasmids bearing the region 1 to 650 (*B*) or 516 to 650 (*C*) were transfected into HEK-293T cells, treated with or without MG132, and then analyzed for MLH1 turnover. *D*, the ubiquitylation profile of two MLH1 fragments. HEK-293T cells were transfected with MLH1 fragments containing 1 to 650 and 516 to 650. Cells treated with or without MG132 were then prepared for the immunoprecipitation and Western blotting assays as indicated. 25% of 1 to 650 fragment sample with MG132-treated or 10% of 516 to 650 fragment sample with MG132-treated was loaded to ensure the amount of MLH1 in DMSO or MG132 samples was equal for a better comparison of the ubiquitylation pattern.

MLH1 is a protein composed of 756 amino acid residues and contains several critical domains (14, 25, 26, 27), including the N-terminal ATPase region, MutS binding segment, and the C-terminal domains for EXO1 and PMS2 interaction (Fig. 2*A*). To delineate the specific sequences responsible for MLH1 turnover, we constructed a serial of MLH1 truncations based on either the functional domains (aa. 1–410, 1–506, 1–650, 1–743) or a disease-associated nonsense mutation (1–516 with Q516∗) (https://www.cbioportal.org/) (Fig. 2*A*). The analysis of the stability of each MLH1 truncation in the presence of CHX showed that the truncations without aa. 516 to 650 were relatively stable, but the truncations include aa. 516 to 650 were quickly degraded (Fig. 2*A*), suggesting that the aa. 516 to 650 region is essential for MLH1 degradation. To confirm this finding, we measured the stability of two representative fragments, aa. 1 to 650 and aa. 516 to 650, in the presence of CHX and MG132. The results showed that MG132 treatment markedly stabilized both fragments (Fig. 2, *B* and *C*). Meanwhile, the ubiquitylation levels of both fragments were increased (Fig. 2*D*). These results suggest that the region encompassing amino acids 516 to 650 might be the degradation signal sequence of MLH1.

### PMS2 and EXO1 promote MLH1 stability

**Figure 3:**
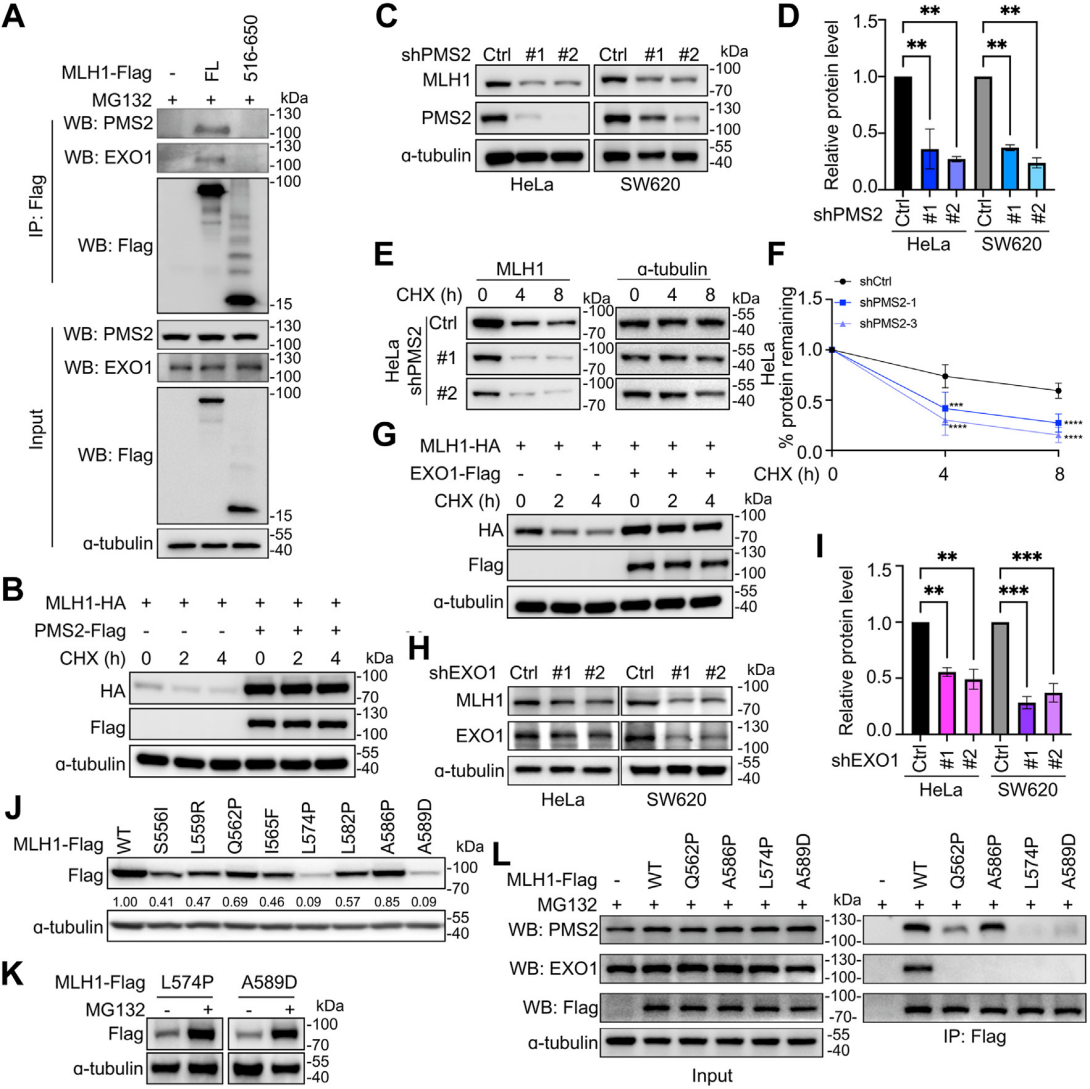
**PMS2 and EXO1 promote MLH1 stability.***A*, the MLH1^516-650^ fragment fails to bind PMS2 and EXO1. HEK-293T cells were transfected with full-length (FL) or the MLH1^516-650^ fragment for 48 h. Cell lysates were then prepared for the immunoprecipitation and Western blotting analysis as indicated. *B*, PMS2 expression blunts MLH1-HA degradation. HEK-293T cells were transfected with MLH1-HA in the absence or presence of PMS2-Flag. Cells were then collected at indicated time points after CHX treatment for degradation analysis. *C*, PMS2 knockdown reduces endogenous MLH1 level. PMS2 was knocked down in HeLa cells with its specific shRNA. Cell lysates were then subjected to the Western blotting assays against MLH1 and PMS2. *D*, quantification of the data of MLH1 steady state in *C* (means ± SD in three independent experiments, *∗∗p* < 0.01, two-tailed Student’s *t* test). *E*, PMS2 knockdown accelerates endogenous MLH1 turnover. HeLa cells with or without PMS2 knockdown were evaluated for MLH1 stability. *F*, quantification of the data of MLH1 protein remaining in *E* (means ± SD in three independent experiments, *∗∗∗p* < 0.001,*∗∗∗∗p* < 0.0001, two-tailed Student’s *t* test). *G*, EXO1 expression impedes MLH1-HA degradation. HEK-293T cells were transfected with MLH1-HA in the absence or presence of EXO1-Flag. Cells were collected at time points as indicated after CHX treatment for MLH1-HA degradation. *H*, EXO1 knockdown enhances endogenous MLH1 turnover. EXO1 was knocked down from cells by infection with its specific shRNA. HeLa cells were collected at indicated time points post-CHX treatment for the analysis of MLH1 turnover. *I,* quantification of the data of MLH1 steady state in *H* (means ± SD in three independent experiments, *∗∗p* < 0.01, *∗∗∗p* < 0.001, two-tailed Student’s *t* test). *J*, steady state levels of MLH1 pathogenic mutants. HEK-293T cells were transfected with MLH1-Flag derivatives for 48 h and then assayed for MLH1 levels. *K*, two MLH1 mutants (L574P, A589D) are regulated by the proteasome. HEK-293T cells that were transfected with MLH1-Flag mutants for 48 h. Cells were then collected in the absence or presence of MG132. *L*, MLH1 mutants exhibit impaired bindings to PMS2 and EXO1. HEK-293T cells were transfected with the empty vector, MLH1-Flag WT, or derivatives for 48 h. MG132 was used to enrich proteins, followed by cell lysate preparation for the IP/Western blot assay as indicated.

The fragment of amino acids 516 to 650 overlaps with the previously mapped regions for binding to EXO1 (aa. 410–650) and PMS2 (aa. 506–743) (27) (Fig. 2*A*). Therefore, we speculated the interaction of MLH1 with PMS and/or EXO1 may play a role in regulating MLH1 stability. To test this hypothesis, we first examined the interaction between full-length MLH1 or the aa. 516 to 650 fragment (MLH1 ^516-650^) and PMS2 or EXO1. Consistent with previous findings, PMS2 and EXO1 co-immunoprecipitated with full-length MLH1 (Fig. 3*A*). However, neither PMS2 nor EXO1 is sufficient to interact with the MLH1 ^516-650^ fragment (Fig. 3*A*). We wondered whether PMS2 or EXO1 promoted the stability of MLH1 as the interaction may mask the region required for MLH1 degradation. Interestingly, ectopic expression of PMS2 impaired MLH1 degradation (Fig. 3*B*). Consistently, the steady state of the MLH1 protein was decreased when PMS2 was knocked down by its specific shRNAs (Fig. 3, *C* and *D*). The degradation rate of MLH1 was enhanced by PMS2 knockdown (Fig. 3, *E* and *F*). Similar results were also observed in cells with EXO1 overexpression or knockdown (Fig. 3, *G*–*I*). These results suggest that PMS2 and EXO1 are required to maintain MLH1 stability.

MLH1 was found with various mutations in many types of cancers, some of which are within the aa. 516 to 650 region, including S556I, L559R, Q562P, I565F, L574P, L582P, A586P, A589D (4, 5, 28, 29, 30, 31, 32). To find out whether these disease-associated mutations affected MLH1 turnover, we assessed the protein levels of the disease mutants. We found that both L574P and A589D were markedly reduced compared to the wild-type MLH1 (Fig. 3*J*). Moreover, these mutants were stabilized by MG132 (Fig. 3*K*). Interestingly, the binding of PMS2 or EXO1 to both L574P and A589D variants was strikingly reduced compared to wild-type MLH1 (Fig. 3*L*). In contrast, Q562P and A586P still bind to PMS2 but lost EXO1 binding while maintaining a steady-state level comparable to wild-type MLH1 (Fig. 3*L*). These results suggest that the interaction with PMS2 but not EXO1 is required for maintaining MLH1 stability.

### The ubiquitin ligase UBR4 promotes MLH1 turnover

**Figure 4:**
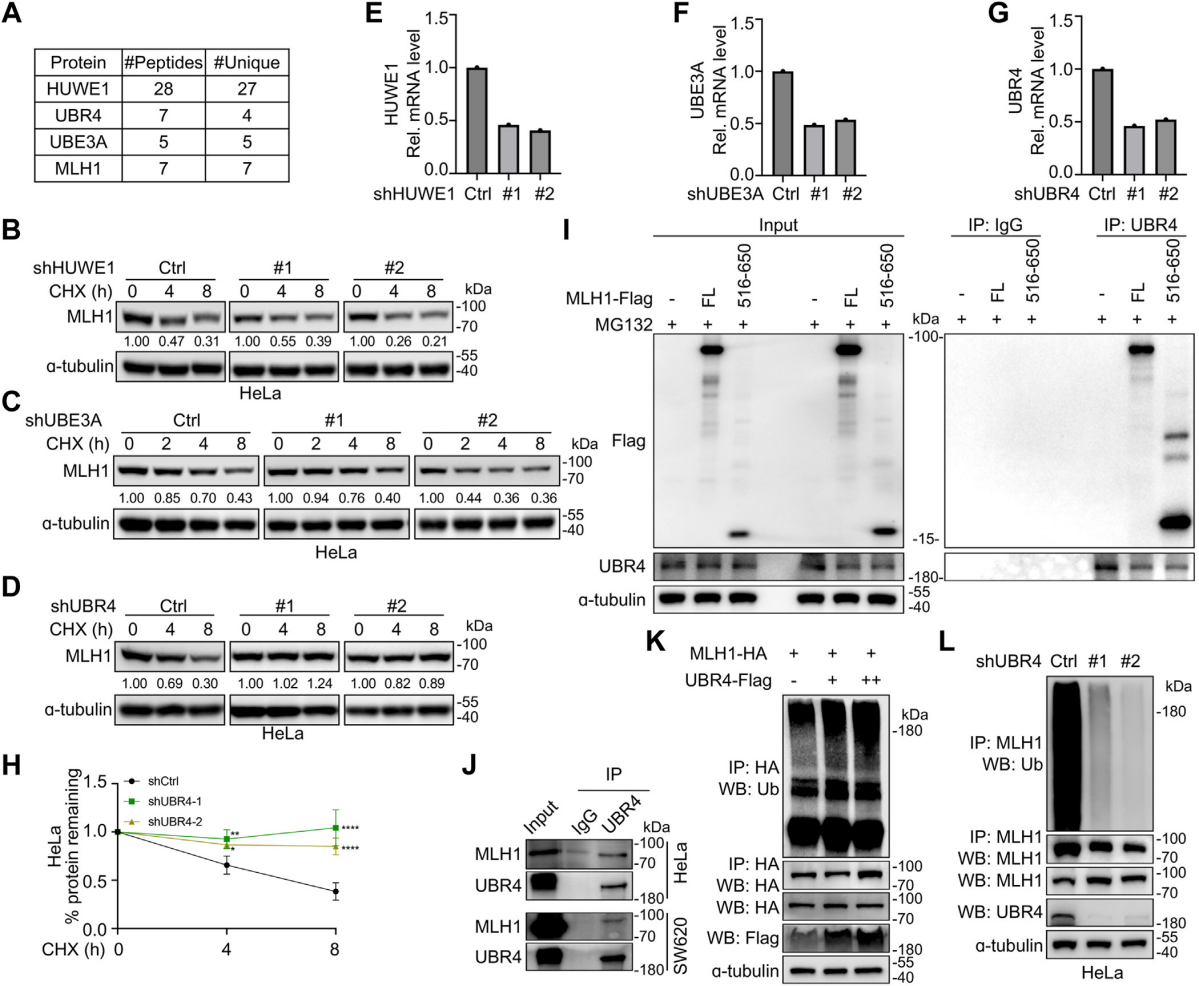
**UBR4 interacts with MLH1 and promotes its ubiquitylation and degradation.***A*, mass spectrometry was performed to identify the E3 candidates of MLH1. HEK-293T cells were transfected with the Flag-tagged MLH1^516-650^ plasmid for 48 h, followed by MG132 treatment for 4 hrs. Whole-cell lysates were then prepared for immunoprecipitation with anti-Flag conjugated magnetic beads. The immunoprecipitated proteins were subjected to HPLC-coupled tandem mass spectrometry. The resultant peptides were subjected to a database search, and three potential E3 ligases identified were shown. *B–G*, MLH1 degradation in cells deficient in one of the three E3s. HeLa cells with HUWE1 (*B* and *E*), UBE3A (*C* and *F*), or UBR4 (*D* and *G*) knockdown were treated with CHX. Cells were harvested at indicated time points after CHX treatment for Western blot and qRT-PCR analyses. *H*, quantification of the data of MLH1 protein remaining in *D* (means ± SD in three independent experiments, *∗p* < 0.05, *∗∗p* < 0.01, *∗∗∗∗p* < 0.0001, two-tailed Student’s *t* test). *I*, region 516 to 650 is critical for UBR4-MLH1 interaction. HEK-293T cells were transfected with empty vector, MLH1^FL^, or MLH1^516-650^ fragment plasmid for 48 h. MG132 was used to enrich proteins. An IP/Western Blot assay was performed, and anti-IgG was used as a negative control. *J*, endogenous UBR4 interacts with MLH1. HeLa and SW620 cells were harvested and lysed. Protein extracts were subjected to immunoprecipitation and Western blotting analysis as indicated to detect the interaction between UBR4 and MLH1. *K*, knockdown of UBR4 blocks MLH1 ubiquitylation. HeLa cells stably expressing indicated shRNA were harvested to prepare for cell lysates, followed by the immunoprecipitation and Western blot assay as indicated to measure the ubiquitylation level of MLH1. *L*, overexpression of UBR4 increases MLH1 ubiquitylation. HEK-293T cells were transfected with UBR4 and MLH1 plasmids for 48 h, followed by cell lysate preparation to measure MLH1 ubiquitylation level.

The rate-limiting component of the ubiquitin-mediated substrate degradation is an E3 ubiquitin ligase that can select and bind to specific targets for ubiquitylation and subsequent degradation. To determine which E3 ligase is responsible for MLH1 ubiquitylation and degradation, we utilized the MLH1^516-650^ fragment as the bait to search for potential ubiquitin ligases by affinity-purification coupled tandem mass spectrometry. Several candidate ubiquitin ligases were identified by mass spectrometry in the MLH1^516-650^-interactome, including HUWE1, UBE3A, and UBR4 (Fig. 4*A*). To find out which E3 might indeed direct MLH1 ubiquitylation and degradation, we knocked down each candidate E3 by its specific shRNA carried by lentiviruses, followed by the measurement of MLH1 degradation rates in the presence of CHX. The results showed that the half-life of MLH1 was not significantly altered when either HUWE1 (Figs. 4, *B* and *E*, and S1*A*) or UBE3A was knocked down (Figs. 4, *C* and *F* and S1*B*). However, MLH1 was strikingly stabilized when UBR4 was knocked down by specific shRNAs (Fig. 4, *D*, *G*, and *H*), suggesting that UBR4 might be an E3 ubiquitin ligase of MLH1 and promote its degradation.

To confirm the interaction between MLH1 and UBR4, the MLH1^FL^ or the MLH1^516-650^ were transfected into HEK-293T cells, followed by the co-immunoprecipitation (co-IP) and Western blot assays. As shown in Figure 4*I*, UBR4 was found to interact with MLH1^FL^ and MLH1^516-650^, suggesting aa. 516 to 650 is a critical region for UBR4-MLH1 interaction. The interaction between endogenous MLH1 and UBR4 was also confirmed in both cervical and colorectal cancer cells (Fig. 4*J*). Furthermore, the more UBR4 was overexpressed, the higher level of the polyubiquitylation of MLH1 was observed (Fig. 4*K*). In contrast, the levels of MLH1 ubiquitylation in shUBR4 cells were strikingly reduced compared to the control cells (Fig. 4*L*). Therefore, all these results suggest that UBR4 is an E3 ubiquitin ligase of MLH1 and directs MLH1 degradation in the proteasomes.

### Deubiquitylase USP5 facilitates MLH1 deubiquitylation

**Figure 5:**
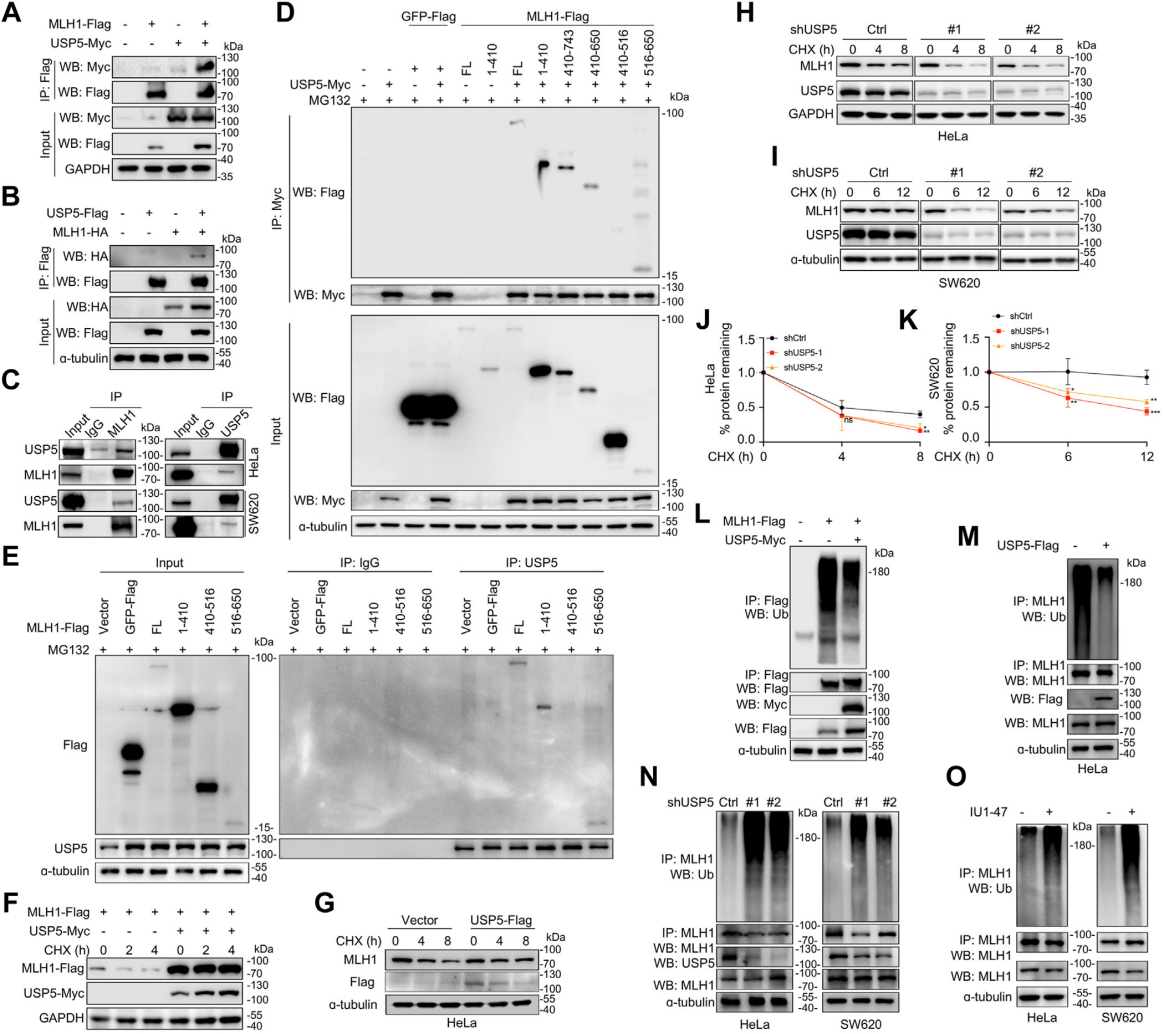
**USP5 interacts with MLH1 and promotes its deubiquitylation and stabilization.***A* and *B*, the interaction between ectopically expressed MLH1 and USP5. HEK-293T cells were transfected with the MLH1 and USP5 plasmids for 48 h, followed by cell lysate preparation and the immunoprecipitation assays to examine the interaction between USP5 and MLH1 as indicated. *C*, endogenous MLH1 binds USP5. Whole-cell extracts from HeLa and SW620 cells were subject to reciprocal immunoprecipitation assays as indicated to detect the MLH1-USP5 association. *D–F*, region 1 to 410 and 516 to 650 were responsible for MLH1-USP5 interaction. HEK293T cells were transfected with USP5-Myc and MLH1-Flag FL or truncated variants plasmids for 48 h. MG132 was used to enrich proteins. Cells were then harvested, and an IP/Western blot assay was performed. *E*, HEK293T cells were transfected with MLH1-Flag FL or truncate plasmids for 48 h. MG132 was used to enrich proteins. Cells were then harvested, and an IP/Western blot assay was performed. *F*, USP5 expression promotes the stability of MLH1-Flag. HEK-293T cells were transfected with the MLH1 and USP5 plasmids for 48 h. Cells were then collected at indicated time points after CHX treatment to monitor MLH1-Flag turnover. *G*. endogenous MLH1 is degraded slower upon USP5 overexpression in HeLa cells. *H–K*, endogenous MLH1 turnover is accelerated upon USP5 knockdown. HeLa (*H*) and SW620 (*I*) cells with USP5 knockdown were treated with CHX for indicated periods. Cells were harvested, followed by Western blot analysis. *J* and *K*, quantifications of the data of MLH1 protein remaining in *H* and *I,* respectively (means ± SD in three independent experiments, *ns* > 0.05, *∗p* < 0.05, *∗∗p* < 0.01, *∗∗∗p* < 0.001, two-tailed Student’s *t* test). *L*, MLH1-Flag ubiquitylation is reduced by USP5 overexpression. HEK-293T cells were co-transfected with MLH1-Flag and USP5-Myc for 48 h. Cell lysates were then prepared for MLH1-Flag ubiquitylation analysis. *M*, endogenous MLH1 ubiquitylation is reduced by USP5 overexpression in HeLa cells. *N*, endogenous MLH1 ubiquitylation is enhanced upon USP5 knockdown. Lentiviral shUSP5 was subject to infect HeLa and SW620 cells. Cell lysates were then prepared to measure MLH1 ubiquitylation. *O*, endogenous MLH1 ubiquitylation is increased by USP5 inhibition. HeLa and SW620 cells were treated with IU1-47, a selective inhibitor of USP5, for 24 h, followed by cell lysate preparation for MLH1 ubiquitylation.

As protein ubiquitylation is a dynamic and reversible process, the conjugated ubiquitin molecules can be clipped off by a deubiquitylase (DUB) (33, 34). We wondered whether MLH1 may be regulated by a DUB. By searching a database (https://thebiogrid.org), we found that a deubiquitylase USP5 may be associated with MLH1. To test this possibility, we performed the co-immunoprecipitation assay and found that MLH1 is associated with both ectopic expressed or endogenous USP5 (Fig. 5, *A*–*C*). To confirm this binding and find out the MLH1 binding region with USP5, several MLH1 truncations were constructed. Each construct was co-transfected with the USP5 plasmids, followed by a co-IP assay. As shown in Figure 5*D*, USP5 interacted with the full-length MLH1 and the aa. 1 to 410 and aa. 516 to 650 fragments but barely interacted with the aa. 410 to 516 fragment. This finding was also confirmed in endogenous USP5 (Fig. 5*E*), suggesting aa. 1 to 410 and 516 to 650 regions are responsible for USP5-MLH1 interactions. Consistent with the interactions between MLH1 and USP5, overexpression of the ubiquitylase USP5 impeded the degradation of both exogenous and endogenous MLH1 (Fig. 5, *F* and *G*). The degradation rate of endogenous MLH1 was increased when USP5 was knocked down (Fig. 5, *H*–*K*). Moreover, USP5 overexpression markedly reduced the ubiquitylation level of both exogenous and endogenous MLH1 (Fig. 5, *L* and *M*). In contrast, USP5 knockdown led to increased ubiquitylation on MLH1 (Fig. 5*N*). Furthermore, MLH1 ubiquitylation was also significantly increased when USP5 was inhibited by its specific inhibitor IU1-47 (Fig. 5*O*). These results collectively support that USP5 is a DUB of MLH1 to deubiquitinate and stabilize MLH1.

### UBR4 or USP5 deficiency affects cellular sensitivity to 6-TG oppositely

**Figure 6:**
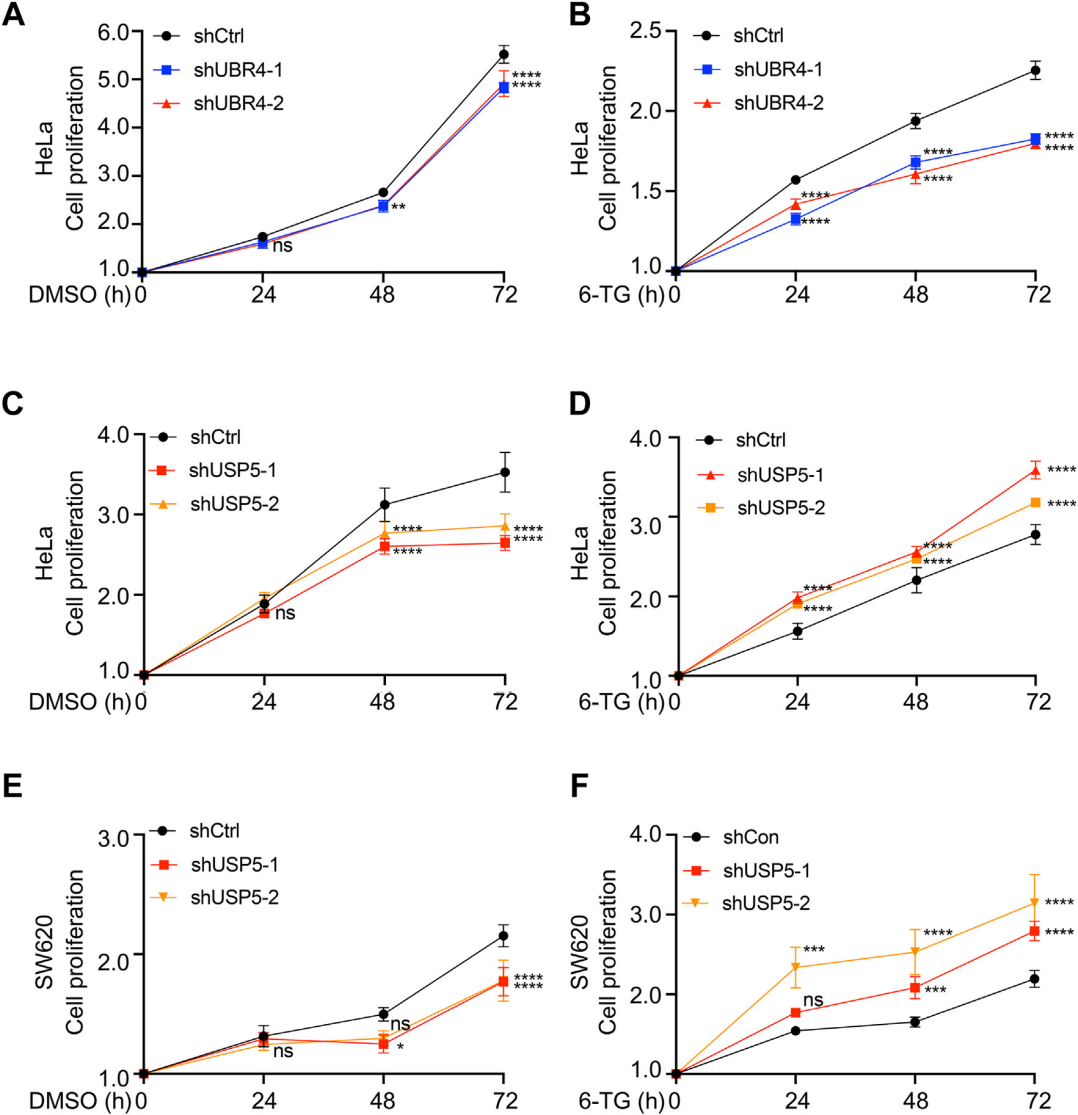
**Knockdown of UBR4 or USP5 alters the sensitivity to 6-TG, a genotoxic agent.***A* and *B*, UBR4 knockdown reduces cell proliferation in the presence of 6-TG. HeLa cells with UBR4 knockdown were treated with DMSO (*A*) or 6-TG (*B*) for indicated periods. *C*–*F*, USP5 knockdown affected cell proliferation in the presence of 6-TG. USP5 was knocked down by its shRNAs in HeLa (*C*–*D*) and SW620 (*E* and *F*) cells, followed by DMSO (*C* and *E*) or 6-TG (*D* and *F*) treatment and CCK-8 assay. All quantifications show the means ± SD in three independent experiments, *ns*>0.05, *∗p* < 0.05, *∗∗p* < 0.01, *∗∗∗p* < 0.001, *∗∗∗∗p* < 0.0001, two-tailed Student’s *t* test.

As an essential component of the mismatch repair pathway, MLH1 depletion leads to MMR dysfunction (1, 7, 10, 26, 35). The nucleotide analog 6-thioguanine (6-TG) can induce mismatched base pairs and is widely used in MMR studies (17, 19, 20). Given that UBR4 and USP5 modulate MLH1 stability, we wondered whether the alteration of UBR4 and USP5 could affect the cellular response to DNA damage induced by 6-TG. As demonstrated previously, we adopted cell viability as a readout of the reaction (17, 19, 20). We knocked down UBR4 or USP5 in HeLa or SW620 cells, followed by 6-TG treatment and the measurement of cell viability using CCK-8 assays, which count the number of living cells based on the amount of formazan dye generated by dehydrogenases (36). The proliferation of cells with UBR4 knockdown was slightly slower than control cells (Fig. 6*A*), consistent with a previous report (37). However, the proliferation of cells with UBR4 knockdown was much slower than that of control cells upon 6-TG treatment (Fig. 6*B*). The data suggests that UBR4 knockdown enhances cellular sensitivity to 6-TG. The proliferation of USP5 knockdown cells was slower than the control cells (Fig. 6, *C* and *E*), as previously reported (38, 39). Interestingly, the USP5 knockdown cells were more tolerant to 6-TG than control cells (Fig. 6, *D* and *F*). Therefore, UBR4 and USP5 deficiency affects the cellular sensitivity to 6-TG oppositely.

## Discussion

MMR is a highly concerted process that requires MutS to recognize the DNA mismatch and, together with MutL, initiates a precise cleavage at the nascent strand of DNA. The MutS complex includes the MutSα (MSH2-MSH6) and MutSβ (MSH2-MSH3) complexes, while the MutL complex includes MutLα complex (MLH1-PMS2) and MutLβ complex (MLH1-MLH3) (6, 8, 9, 13, 37, 38). MMR activity can be adjusted through the regulation of each essential component, including MLH1, MSH2, *etc.* MLH1 is known to be regulated at the transcriptional level (40, 41, 42, 43, 44). Under hypoxia conditions, two transcription repressors, DEC1 and DEC2, are induced and bind to E-box-like motif(s) in the MLH1 promoter region to inhibit its expression, compromising the MMR system (40). MLH1 is highly regulated post-translationally as well. Casein kinase II (CK2) is identified to phosphorylate MLH1 at Serine 477, leading to the dysfunction of MMR (16). Likewise, the phosphorylation at Serine 87 of MLH1 inhibits its DNA binding (45). Furthermore, the thermal stability of MutLα is decreased in correlation with its phosphorylation status (45). Interestingly, HDAC6 is reported to deacetylate MLH1, disassembling the MutS and MutL complexes (18). A common phenomenon in these studies is that MLH1 down-regulation, therefore, MMR deficiency results in tolerance to 6-TG in cells. Besides MLH1, the MutSα component is also regulated at the post-translational level. An unexpected E3 ligase activity of HDAC6 is reported to regulate MSH2 degradation by the proteasomes (17), whereas USP10 is demonstrated as the DUB for MSH2, protecting it from degradation (20). Accordingly, the knockdown of USP10 promotes cell survival and inhibits apoptosis upon the treatment of genotoxic agents MNNG and 6-TG due to MSH2/MMR deficiency.

Interestingly, unbalanced levels of MutL subunits cause defects in the MMR (21, 46). Overexpression of either MLH1 or PMS2 leads to hyper-mutagenesis in yeast cells, while co-expression of its partner can alleviate the highly increased mutations (21). It suggests that an orphan MutL protein can interfere with the MMR process, and cells need to keep the right level of each MutL protein for the sake of functional MMR. They are probably required to be kept in the right concentration and carefully balanced through ubiquitylation and deubiquitylation. Previous studies, and we have found that MLH1 and PMS2 always coexist in cells (28, 47), but no cases where cells contain only MLH1 or PMS2 protein at an average level have been reported. In the absence of PMS2, the MLH1 level is extremely low, albeit the underlying mechanism remains unclear. In the present study, we found that the interactions between PMS2 and MLH1 promote MLH1 stability (Fig. 3). It is not unusual that an “orphan” protein is unstable and becomes stabilized upon finding a binding partner. This is likely the case for MLH1 as its degradation signal sequence (aa. 516–650) is involved in the binding with PMS2.

We found that MLH1 is processed *via* the ubiquitin-proteasome fine-tuned by the E3 ligase UBR4 and the DUB USP5. Interestingly, UBR4 interacts with MLH1 through a.a. 516 to 650 region. It suggests that PMS2 can cover aa. 516 to 650 region of MLH1 to shield it from ubiquitylation by UBR4.

MLH1 contains four major domains: the N-terminal ATPase region, the MSH2 binding domain, and the C-terminal segments for binding to EXO1 and PMS2 (8, 9, 27, 48). MLH1 is involved in DNA end processing with the ATPase domain during the class switch recombination (48). MLH1 brings PMS2 and EXO1 to the lesions for downstream processing (49, 50, 51, 52). Interestingly, a recent study found that the MLH1-EXO1 interaction restricts EXO1 function. In the absence of MLH1, EXO1 displays excessive exonuclease activity, leading to more DNA breaks (53). Many pathogenic mutations lie in the aa. 516 to 650 region, the degradation signal of MLH1. However, the nature of these mutants and how they contribute to the onset of diseases is unknown. We examined the EXO1 and PMS2 interactions for four of these mutants. Interestingly, Q562P and A586P mutants maintain the interaction with PMS2 and have comparable steady-state levels as wild-type MLH1. In contrast, the L574P and A589D mutants lost the interaction with PMS2 and are low at the steady state. Intriguingly, all four mutants abolished their interactions with EXO1. These data suggest that PMS2 interaction is critical for MLH1 stability while EXO1 interaction is not. However, overexpression or knockdown of Exo1 also affects MLH1 stability, likely through an indirect yet unknown mechanism that is different from how PMS2 promotes MLH1 stability. It will be interesting to test whether Q562P and A586P pathogenesis rely on unrestrained EXO1 activity and DNA breaks formation.

We have identified essential cis-elements and trans-factors critical for MLH1 degradation, revealing a novel mechanism for regulating MMR activity. Interestingly, UBR4 and USP5 both impinge on MLH1 and have an opposite effect in 6-TG resistance without altering the steady-state level of MLH1, indicating their more direct roles in MMR. Further mechanistic elucidation is needed to understand how they are involved in MMR. One possibility is that MutL needs to be dissembled/degraded by proteasome once MMR is complete. It might also be interesting to investigate whether the UBR4/USP5/MLH1 or EXO1/MLH1 axis regulates heteroduplex rejection during homologous recombination in mammalian cells. Our study provides novel insights into the physiological significance of UBR4-regulated MLH1 turnover and a new anti-cancer strategy avenue.

## Experimental procedures

### Cell culture

All cell lines were cultured in Dulbecco’s modified Eagle’s Medium (DMEM) containing 10% fetal bovine serum, penicillin (100 U/ml), and streptomycin (100 g/ml). All these agents were provided by Thermal Fishier Life Technologies. Cells were incubated at 37 °C with 5% CO_2_.

### Antibodies and chemicals

Antibodies used, including anti-PMS2 (Cat. #ab110638) and anti-UBR4 (Cat. #ab86738), were purchased from Abcam. The anti-Flag antibody (Cat. #M8823) was purchased from Sigma-Aldrich. The anti-HA (Cat. #2999S) and the anti-Myc antibodies (Cat. #2040S) were provided by Cell Signaling Technology. The anti-GAPDH (Cat. #HRP-60004), the anti-ɑ-tubulin (Cat. #HRP-66031), the anti-ubiquitin (Cat. #80992-1-RR), and the anti-USP5 antibodies (Cat. #10473-1-AP) were purchased from Proteintech. The anti-EXO1 antibody (Cat. #MA512262) was purchased from Invitrogen. Reagents, MG132 (Cat. #HY-13259), IU1-47 (Cat. #HY-122243), and Cycloheximide (Cat. #HY-12320) were obtained from MedChemExpress. 6-TG (Cat. #A4882) was purchased from Sigma-Aldrich.

### Cycloheximide (CHX) chase assay

Cells were treated with 50 μ g/ml of CHX for specific periods before being collected for cell lysate preparation and Western blot assays as described previously (54).

### Plasmids

The plasmids MLH1 with Flag-tag, HA-tag, and MLH1 truncates were obtained from WZ Biosciences. The plasmids PMS2-Flag, EXO1-Flag, and USP5-Myc were purchased from Han Yi Biosciences. The plasmid UBR4-Flag was a generous gift from Dr Baotong Zhang (Southern University of Science and Technology). The USP5 plasmid was constructed as described previously (55).

### Construction of MLH1 variants

A series of MLH1 disease mutations were amplified by Q5 Site-Directed Mutagenesis Kit (Cat. #E0552S, New England Biolabs) based on the MLH1-Flag plasmid. Primers synthesized by Sangon Biotech were shown in Table 1.

**Table 1:** The primers for MLH1 disease mutation construction

Mutants	Direction	Sequences (5′-3′)
S556I	Forward	AAGCTTATTGAAGAACTGTTCTACCAG
	Reverse	GGTGGTGTTGAGAAGGTATAACTTGGT
L559R	Forward	TGAAGAACGGTTCTACCAGATACTCATTTATGATTTTGC
	Reverse	CTAAGCTTGGTGGTGTTGAGAAGGTATAAC
Q562P	Forward	TTCTACCCGATACTCATTTATGATTTT
	Reverse	CAGTTCTTCACTAAGCTTGGTGGTGTT
I565F	Forward	ATACTCTTTTATGATTTTGCCAATTTT
	Reverse	CTGGTAGAACAGTTCTTCACTAAGCTT
L574P	Forward	GGTGTTCCCAGGTTATCGGAGCCAGCA
	Reverse	AAAATTGGCAAAATCATAAATGAGTAT
L582P	Forward	GCACCGCCCTTTGACCTTGCCATGCTT
	Reverse	TGGCTCCGATAACCTGAGAACACCAAA
A586P	Forward	GACCTTCCCATGCTTGCCTTAGATAGT
	Reverse	AAAGAGCGGTGCTGGCTCCGATAACCT
A589D	Forward	ATGCTTGACTTAGATAGTCCAGAGAGT
	Reverse	GGCAAGGTCAAAGAGCGGTGCTGGCTC

### Lentiviral shRNA and establishment of stable knockdown cell lines

To generate lentiviral shRNA, the pLKO.1 vector containing shRNA of interest was co-transfected with psPAX2 and pMD2.G into HEK-293T cells for lentivirus packaging. After 48 h of transfection, the lentiviral particles in the cell medium were harvested. Then, the mixed solution (lentivirus particle 1:1 fresh total medium and polybrene (10 mg/ml stock, 1:1000)) was added into cells. About 6 h after infection, a fresh medium with 10% FBS, replacing the mixed solution, was added to cells. 24 ∼ 36 h later, 1 μ g/ml puromycin was added into the medium to select the cells. After the selection of puromycin for 2 weeks, Western blot analyses were used to determine the knock-down efficiency. Lentivirus-delivered shRNA oligos against indicated genes were purchased from Sangon Biotech. The shRNA target oligos were listed in Table 2.

**Table 2:** Oligos for shRNA

Target	Sequence (5′-3′)
Control	TTCTCCGAACGTGTCACGT
PMS2-1	CCAGGAAGATACCGGATGTAA
PMS2-2	CGTGTGTGAAGAGTACGGTTT
EXO1	GAACAAGGTTCCTGGGCTATA
HUWE1-1	CCACACTTTCACAGATACTAT
HUWE1-2	GCTCCCACTATAACCTCACTT
UBE3A-1	CCTACATCTCATACTTGCTTT
UBE3A-2	CGGAATACTCAAGCAAAGAAA
UBR4-1	CCACATACATTGTTCGGGAAA
UBR4-2	CCACCATCAAAGACTTACATT
USP5-1	GACCACACGATTTGCCTCATT
USP5-2	GATAGACATGAACCAGCGGAT

### Immunoprecipitation (IP)

Cells of interest were lysed in cold IP lysis buffer (50 mM Heps, 150 mM NaCl, 10% Glycerol, 1% TritonX-100, and 1 mM EDTA) (3, 54). After clarification in the cold with high-speed centrifugation, the clear supernatants were pre-incubated with Protein G beads (Cat. #10004D, Thermo Fisher Scientific) for 2 h. After a short spin down, the supernatants were then incubated with primary antibodies indicated for 12 h at 4 °C. Protein G beads were used to immunoprecipitate the primary antibodies for 4 ∼ 6 h at 4 °C before being collected for subsequent mass spectrometry or Western blotting assays.

### *In vivo* ubiquitylation assay

Denatured IP was used to measure the ubiquitylation level described in the previous work (56). Briefly, cells were harvested and lysed with SDS-denaturing buffer (62.5 mM Tris-HCl pH 6.8, 2% SDS, 10% glycerol, 1.5% β-mercaptoethanol). Ultrasonic was used to lyse further before the samples were boiled for 10 min. Native lysis buffer (50 mM Tris-HCl pH 7.4, 0.5% Triton X-100, 200 mM NaCl, 10% glycerol) was used to dilute the cell sample tenfold to fortyfold. After centrifugation at 13,000 rpm for 5 min, the supernatants were immunoprecipitated using antibodies of interest at 4 °C for 1 h. Native lysis buffer was used to wash the immunocomplexes three times, resolved using SDS–PAGE, and immunoblotted against indicated antibodies.

### Affinity-purification coupled tandem mass spectrometry (AP/MS/MS)

The AP/MS/MS was conducted as described previously (54, 55, 57). Basically, cells expressing the degron (aa. 516–650) with Flag tag were treated with MG132 (10 μM) for 4 h and lysed with a lysis buffer as described previously (54). After clarification, 10 mg proteins were first pre-incubated with Protein A+G beads for 1 h at room temperature. The supernatants were mixed with anti-Flag beads overnight at 4 °C. Subsequent procedures were described previously (54). After stringent washes, the beads were subjected to mass spectrometry for analysis (54). Candidate ubiquitin ligases were chosen from each MS analysis because more than two unique peptides were identified with confidence >95%.

### Quantitative real-time PCR

Total RNA was prepared using the RNA Isolation Kit (Cat. # RC112, Vazyme), followed by cDNA synthesis using RT SuperMix for the qPCR Kit (Cat. #R323, Vazyme). qRT-PCR was carried out using Taq Pro Universal SYBR qPCR Master Mix (Cat. #Q712, Vazyme) with a Step One PlusTM real-time PCR system (Thermo Fisher Scientific). The primers used for qRT-PCR are listed in Table 3.

**Table 3:** Primers for qRT-PCR

Gene	Forward primers	Reverse primers
GAPDH	GGAGCGAGATCCCTCCAAAAT	GGCTGTTGTCATACTTCTCATGG
HUWE1	CAAACTACATCACTCGTCTGGG	AGTCTCTGCAACATTCTGCAAG
UBE3A	CTCAGCTTACCTTGAGAACTCG	TTCTAGCGCCTTTCTTGTTCAT
UBR4	TCCTACTCCGCCTTCGAGATG	CTGAAGTTGGTTCCGGGGAAT

### Cell proliferation assay

CCK8 allows convenient assays using WST-8 (2-(2-methoxy-4-nitrophenyl)-3-(4-nitrophenyl)-5-(2,4-disulfophenyl)-2H-tetrazolium, monosodium salt), which produces a water-soluble formazan dye upon reduction in the presence of an electron carrier, 1-Methoxy PMS. A microplate reader is used to measure the Optical Density at 450 nm. The measurements can indirectly show the number of living cells. Cell Counting Kit (CCK-8) (Cat. #40203ES80, Yeasen Biotechnology) was used to detect cell proliferation and viability. The protocol of Yeasen Biotechnology was followed. Briefly, the cells (100 μ L/well) were plated in a 96-well plate, and the plate was incubated at 37 °C with 5% CO2 for 24 h for pre-incubation. 10 μ L of different concentrations or kinds of the substance was added to the plate, and the plate was incubated for a certain period, such as 0, 24, 48, or 72 h). Then, 10 μ L of CCK-8 reagent was added to each well and mixed gently. The plate was incubated for 1 to 4 h, and a microplate reader was used to measure the absorbance of the plate at 450 n M.

### Statistics

All experiments were performed at least thrice, and the results were presented as mean ± SD. GraphPad Prism V8.0 (GraphPad Software) was employed for statistical analysis. Two-tailed Student’s *t* test was performed to determine statistically significant differences between groups. *ns* > 0.05, *∗p* < 0.05, *∗∗p* < 0.01,*∗∗∗p* < 0.001, *∗∗∗∗p* < 0.0001. *p* < 0.05 was considered statistically significant.

## Data availability

All data that support this study are provided in the article.

## Supporting information

This article contains supporting information.

## Conflict of interest

The authors declare that they have no conflicts of interest with the contents of this article.

## References

[1] Li Z., Pearlman A.H., Hsieh P. (2016). DNA mismatch repair and the DNA damage response. DNA Repair (Amst).

[2] Ijsselsteijn R., Jansen J.G., de Wind N. (2020). DNA mismatch repair-dependent DNA damage responses and cancer. DNA Repair (Amst).

[3] He Y., Jiang S., Mao C., Zheng H., Cao B., Zhang Z. (2021). The deubiquitinase USP10 restores PTEN activity and inhibits non-small cell lung cancer cell proliferation. J. Biol. Chem..

[4] Raevaara T.E., Korhonen M.K., Lohi H., Hampel H., Lynch E., Lönnqvist K.E. (2005). Functional significance and clinical phenotype of nontruncating mismatch repair variants of MLH1. Gastroenterology.

[5] Wanat J.J., Singh N., Alani E. (2007). The effect of genetic background on the function of Saccharomyces cerevisiae mlh1 alleles that correspond to HNPCC missense mutations. Hum. Mol. Genet..

[6] Edelbrock M.A., Kaliyaperumal S., Williams K.J. (2013). Structural, molecular and cellular functions of MSH2 and MSH6 during DNA mismatch repair, damage signaling and other noncanonical activities. Mutat. Res..

[7] Pećina-Šlaus N., Kafka A., Salamon I., Bukovac A. (2020). Mismatch repair pathway, genome stability and cancer. Front. Mol. Biosci..

[8] Ban C., Yang W. (1998). Crystal structure and ATPase activity of MutL: implications for DNA repair and mutagenesis. Cell.

[9] Ellison A.R., Lofing J., Bitter G.A. (2004). Human MutL homolog (MLH1) function in DNA mismatch repair: a prospective screen for missense mutations in the ATPase domain. Nucleic Acids Res..

[10] Fishel R. (2015). Mismatch repair. J. Biol. Chem..

[11] Dai J., Sanchez A., Adam C., Ranjha L., Reginato G., Chervy P. (2021). Molecular basis of the dual role of the Mlh1-Mlh3 endonuclease in MMR and in meiotic crossover formation. Proc. Natl. Acad. Sci. U. S. A..

[12] Rogacheva M.V., Manhart C.M., Chen C., Guarne A., Surtees J., Alani E. (2014). Mlh1-Mlh3, a meiotic crossover and DNA mismatch repair factor, is a Msh2-Msh3-stimulated endonuclease. J. Biol. Chem..

[13] Cannavo E., Sanchez A., Anand R., Ranjha L., Hugener J., Adam C. (2020). Regulation of the MLH1-MLH3 endonuclease in meiosis. Nature.

[14] Abildgaard A.B., Stein A., Nielsen S.V., Schultz-Knudsen K., Papaleo E., Shrikhande A. (2019). Computational and cellular studies reveal structural destabilization and degradation of MLH1 variants in Lynch syndrome. Elife.

[15] Tamura K., Kaneda M., Futagawa M., Takeshita M., Kim S., Nakama M. (2019). Genetic and genomic basis of the mismatch repair system involved in Lynch syndrome. Int. J. Clin. Oncol..

[16] Weßbecher I.M., Hinrichsen I., Funke S., Oellerich T., Plotz G., Zeuzem S. (2018). DNA mismatch repair activity of MutLα is regulated by CK2-dependent phosphorylation of MLH1 (S477). Mol. Carcinog..

[17] Zhang M., Xiang S., Joo H.Y., Wang L., Williams K.A., Liu W. (2014). HDAC6 deacetylates and ubiquitinates MSH2 to maintain proper levels of MutSα. Mol. Cell.

[18] Zhang M., Hu C., Moses N., Haakenson J., Xiang S., Quan D. (2019). HDAC6 regulates DNA damage response via deacetylating MLH1. J. Biol. Chem..

[19] Wu Q., Huang Y., Gu L., Chang Z., Li G.M. (2021). OTUB1 stabilizes mismatch repair protein MSH2 by blocking ubiquitination. J. Biol. Chem..

[20] Zhang M., Hu C., Tong D., Xiang S., Williams K., Bai W. (2016). Ubiquitin-specific peptidase 10 (USP10) deubiquitinates and stabilizes MutS homolog 2 (MSH2) to regulate cellular sensitivity to DNA damage. J. Biol. Chem..

[21] Shcherbakova P.V., Hall M.C., Lewis M.S., Bennett S.E., Martin K.J., Bushel P.R. (2001). Inactivation of DNA mismatch repair by increased expression of yeast MLH1. Mol. Cell Biol..

[22] Obrig T.G., Culp W.J., McKeehan W.L., Hardesty B. (1971). The mechanism by which cycloheximide and related glutarimide antibiotics inhibit peptide synthesis on reticulocyte ribosomes. J. Biol. Chem..

[23] Yan Z., Shanmugasundaram K., Ma D., Luo J., Luo S., Rao H. (2020). The N-terminal domain of the non-receptor tyrosine kinase ABL confers protein instability and suppresses tumorigenesis. J. Biol. Chem..

[24] Mauvezin C., Neufeld T.P. (2015). Bafilomycin A1 disrupts autophagic flux by inhibiting both V-ATPase-dependent acidification and Ca-P60A/SERCA-dependent autophagosome-lysosome fusion. Autophagy.

[25] Vilar E., Gruber S.B. (2010). Microsatellite instability in colorectal cancer-the stable evidence. Nat. Rev. Clin. Oncol..

[26] Boland C.R., Goel A. (2010). Microsatellite instability in colorectal cancer. Gastroenterology.

[27] Schmutte C., Sadoff M.M., Shim K.S., Acharya S., Fishel R. (2001). The interaction of DNA mismatch repair proteins with human exonuclease I. J. Biol. Chem..

[28] Belvederesi L., Bianchi F., Loretelli C., Gagliardini D., Galizia E., Bracci R. (2006). Assessing the pathogenicity of MLH1 missense mutations in patients with suspected hereditary nonpolyposis colorectal cancer: correlation with clinical, genetic and functional features. Eur. J. Hum. Genet..

[29] Houlleberghs H., Dekker M., Lusseveld J., Pieters W., van Ravesteyn T., Verhoef S. (2020). Three-step site-directed mutagenesis screen identifies pathogenic MLH1 variants associated with Lynch syndrome. J. Med. Genet..

[30] Fan Y., Wang W., Zhu M., Zhou J., Peng J., Xu L. (2007). Analysis of hMLH1 missense mutations in East Asian patients with suspected hereditary nonpolyposis colorectal cancer. Clin. Cancer Res..

[31] Lucci-Cordisco E., Boccuto L., Neri G., Genuardi M. (2006). The use of microsatellite instability, immunohistochemistry and other variables in determining the clinical significance of MLH1 and MSH2 unclassified variants in Lynch syndrome. Cancer Biomark.

[32] Rath A., Radecki A.A., Rahman K., Gilmore R.B., Hudson J.R., Cenci M. (2022). A calibrated cell-based functional assay to aid classification of MLH1 DNA mismatch repair gene variants. Hum. Mutat..

[33] Neutzner M., Neutzner A. (2012). Enzymes of ubiquitination and deubiquitination. Essays Biochem..

[34] Sun T., Liu Z., Yang Q. (2020). The role of ubiquitination and deubiquitination in cancer metabolism. Mol. Cancer.

[35] Jiricny J. (2013). Postreplicative mismatch repair. Cold Spring Harb. Perspect. Biol..

[36] Zhang J., Ma C., Yu Y., Liu C., Fang L., Rao H. (2023). Single amino acid-based PROTACs trigger degradation of the oncogenic kinase BCR-ABL in chronic myeloid leukemia (CML). J. Biol. Chem..

[37] Nakatani Y., Konishi H., Vassilev A., Kurooka H., Ishiguro K., Sawada J. (2005). p600, a unique protein required for membrane morphogenesis and cell survival. Proc. Natl. Acad. Sci. U. S. A..

[38] Huang W., Liu X., Zhang Y., Deng M., Li G., Chen G. (2022). USP5 promotes breast cancer cell proliferation and metastasis by stabilizing HIF2α. J. Cell Physiol..

[39] Pan J., Qiao Y., Chen C., Zang H., Zhang X., Qi F. (2021). USP5 facilitates non-small cell lung cancer progression through stabilization of PD-L1. Cell Death Dis..

[40] Nakamura H., Tanimoto K., Hiyama K., Yunokawa M., Kawamoto T., Kato Y. (2008). Human mismatch repair gene, MLH1, is transcriptionally repressed by the hypoxia-inducible transcription factors, DEC1 and DEC2. Oncogene.

[41] Yanamadala S., Ljungman M. (2003). Potential role of MLH1 in the induction of p53 and apoptosis by blocking transcription on damaged DNA templates. Mol. Cancer Res..

[42] Chen J., Sadowski I. (2005). Identification of the mismatch repair genes PMS2 and MLH1 as p53 target genes by using serial analysis of binding elements. Proc. Natl. Acad. Sci. U. S. A..

[43] Tou S.I., Drye E.R., Boulos P.B., Hollingsworth S.J. (2004). Activity (transcription) of the genes for MLH1, MSH2 and p53 in sporadic colorectal tumours with micro-satellite instability. Br. J. Cancer.

[44] Stubbert L.J., Smith J.M., McKay B.C. (2010). Decreased transcription-coupled nucleotide excision repair capacity is associated with increased p53- and MLH1-independent apoptosis in response to cisplatin. BMC Cancer.

[45] Firnau M.B., Plotz G., Zeuzem S., Brieger A. (2023). Key role of phosphorylation sites in ATPase domain and Linker region of MLH1 for DNA binding and functionality of MutLα. Sci. Rep..

[46] Engel C., Ahadova A., Seppälä T.T., Aretz S., Bigirwamungu-Bargeman M., Bläker H. (2020). Associations of pathogenic variants in MLH1, MSH2, and MSH6 with risk of colorectal adenomas and tumors and with somatic mutations in patients with lynch syndrome. Gastroenterology.

[47] Hinrichsen I., Weßbecher I.M., Huhn M., Passmann S., Zeuzem S., Plotz G. (2017). Phosphorylation-dependent signaling controls degradation of DNA mismatch repair protein PMS2. Mol. Carcinog..

[48] Chahwan R., van Oers J.M., Avdievich E., Zhao C., Edelmann W., Scharff M.D. (2012). The ATPase activity of MLH1 is required to orchestrate DNA double-strand breaks and end processing during class switch recombination. J. Exp. Med..

[49] D'Arcy B.M., Arrington J., Weisman J., McClellan S.B., Vandana, Yang Z. (2022). PMS2 variant results in loss of ATPase activity without compromising mismatch repair. Mol. Genet. Genomic Med..

[50] Kadyrov F.A., Dzantiev L., Constantin N., Modrich P. (2006). Endonucleolytic function of MutLalpha in human mismatch repair. Cell.

[51] D'Arcy B.M., Blount J., Prakash A. (2019). Biochemical and structural characterization of two variants of uncertain significance in the PMS2 gene. Hum. Mutat..

[52] Jia P., Chastain M., Zou Y., Her C., Chai W. (2017). Human MLH1 suppresses the insertion of telomeric sequences at intra-chromosomal sites in telomerase-expressing cells. Nucleic Acids Res..

[53] Guan J., Lu C., Jin Q., Lu H., Chen X., Tian L. (2021). MLH1 deficiency-triggered DNA hyperexcision by exonuclease 1 activates the cGAS-STING pathway. Cancer Cell.

[54] Zhuang H., Ren Y., Mao C., Zhong Y., Zhang Z., Cao B. (2022). Induction of zinc finger protein RNF6 auto-ubiquitination for the treatment of myeloma and chronic myeloid leukemia. J. Biol. Chem..

[55] Xu Y., Xu M., Tong J., Tang X., Chen J., Chen X. (2021). Targeting the Otub1/c-Maf axis for the treatment of multiple myeloma. Blood.

[56] Zhu G., Herlyn M., Yang X. (2021). TRIM15 and CYLD regulate ERK activation via lysine-63-linked polyubiquitination. Nat. Cell Biol..

[57] Zhang J., Fan X., Zhou Y., Chen L., Rao H. (2022). The PRMT5-LSD1 axis confers Slug dual transcriptional activities and promotes breast cancer progression. J. Exp. Clin. Cancer Res..

