# Supplementary material for: The ubiquitin ligase UBR4 and the deubiquitylase USP5 modulate the stability of DNA mismatch repair protein MLH1
Source: J Biol Chem. 2024 Jul 18;300(8):107592. doi: 10.1016/j.jbc.2024.107592 (PMC11375253; doi:10.1016/j.jbc.2024.107592)
Supplement: Supplemental Figure 1 legend [file mmc1.docx]

**Supplemental Figure 1. Knockdown of HUWE1 or UBE3A had no significant effect on MLH1 stability.** *A-B*, quantifications of the data of MLH1 protein remaining in Fig 4*B* and *C*， respectively (means ± SD in three independent experiments, ns>0.05, **p*<0.05, two-tailed Student’s *t*-test).
