# Supplementary figures and images for: The ubiquitin ligase UBR4 and the deubiquitylase USP5 modulate the stability of DNA mismatch repair protein MLH1
Source: J Biol Chem. 2024 Jul 18;300(8):107592. doi: 10.1016/j.jbc.2024.107592 (PMC11375253; doi:10.1016/j.jbc.2024.107592)

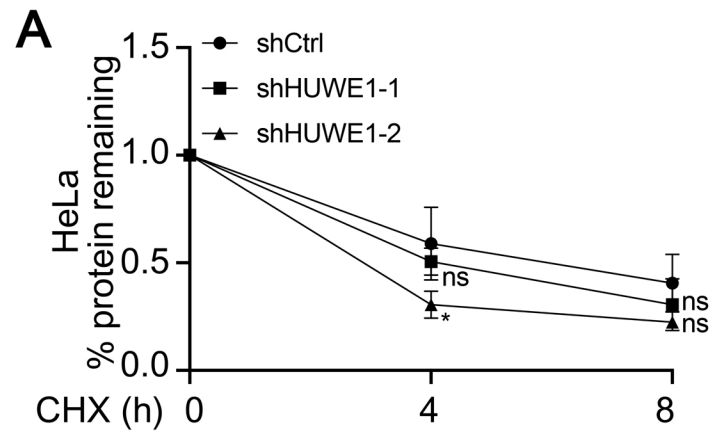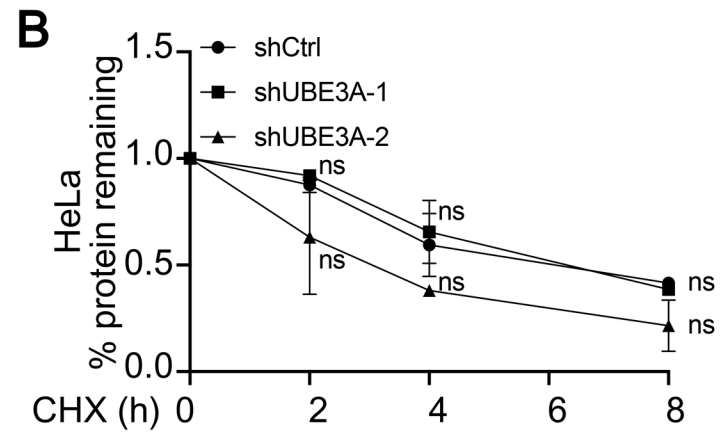

Supplemental Figure 1

Supplement: Supporting information Figure 1 [file mmc2.pdf]
